# Pains and Anodynes

**Published:** 1881-07

**Authors:** 


### PAINS AND ANODYNES.

Dr. Roberts Bartholow, of Philadelphia, says : Several elements enter into the composition of pain—the peripheral irritation, the transmission of the impression to the centre, and its realization by consciousness. Hence, pain may be relieved either by interrupting its transmission to the centres of conscious impressions, or by suspending the functions of these centres. For example, aconite and gelseminum relieve pain in the former manner, and the anæsthetics in the latter. The anæsthetics, when applied locally, however, have an effect similar to aconite, and are, therefore, antagonistic to both peripheral and centric neuralgia. When a few minims of chloroform are injected into the neighborhood of a nerve-trunk, the peripheral expansion of the nerve is put into an anæsthetic and analgesic condition ; and since he has introduced this method of treating sciatica, cervico-brachial and intercostal neuralgia, coccydynia, and other neuralgias of nerves in accessible situations, his experience has been extremely satisfactory. The needle must be inserted deeply, since merely to inject chloroform under the skin, like morphia, is perfectly useless in such neuralgias, unless the nerve-trunk is in the immediate vicinity. No danger attends this expedient, and inflammatory induration and abscesses very rarely result from it. The most powerful means for relief of pain which is now in our possession—the subcutaneous injection of morphia and atropia together—is an illustration of the advantages derived from the study of physiological antagonism. By this combination the anodyne qualities of the two agents are enhanced, rather than diminished, while the disadvantages of each are in a great measure obviated. The combined use of morphia and atropia is, also, the best preventive of the tendency of anæsthetics, like chloroform and ether, to produce fatal paralysis of the heart or lungs; while the prescription of atropia simultaneously with chloral to a great extent averts the dangers that sometimes attend the use of that agent.—*Nashville Jour. of Med. Surg.*, Feb., 1881.

